# Associations Between Anxiety and Home Learning Difficulties in Children and Adolescents with ADHD During the COVID-19 Pandemic

**DOI:** 10.1007/s10578-022-01338-3

**Published:** 2022-03-15

**Authors:** Anna Jackson, Glenn A. Melvin, Melissa Mulraney, Stephen P. Becker, Mark A. Bellgrove, Jon Quach, Delyse Hutchinson, Elizabeth M. Westrupp, Alicia Montgomery, Emma Sciberras

**Affiliations:** 1 Centre for Social and Early Emotional Development, Deakin University, Burwood, VIC Australia; 2 Murdoch Children’s Research Institute, Parkville, VIC Australia; 3 Department of Paediatrics, University of Melbourne, Parkville, VIC Australia; 4 Institute for Social Neuroscience, ISN Innovations, Ivanhoe, VIC Australia; 5 Cincinnati Children’s Hospital Medical Center, Cincinnati, OH USA; 6 University of Cincinnati College of Medicine, Cincinnati, OH USA; 7 Turner Institute for Brain and Mental Health, School of Psychological Sciences, Monash University, Clayton, VIC Australia; 8 Melbourne Graduate School of Education, University of Melbourne, Parkville, VIC Australia; 9 University of New South Wales, Kensington, NSW Australia; 10 Judith Lumley Centre, La Trobe University, Victoria, Australia; 11 Department of Community Paediatrics, Sydney Local Health District, Sydney, NSW Australia

**Keywords:** Attention-deficit/hyperactivity disorder (ADHD), Children, COVID-19, Home learning, Anxiety

## Abstract

The COVID-19 pandemic has markedly impacted functioning for children and adolescents including those with attention-deficit/hyperactivity disorder (ADHD). We explored home learning difficulties (HLD) during COVID-19 restrictions in Australian children (aged 5–17) with ADHD, aiming to: (1) describe home learning experiences, and (2) examine associations between child anxiety (i.e., concurrent anxiety symptoms and pre-existing anxiety disorder status) and HLD. Baseline data from the longitudinal ADHD COVID-19 Survey were used (*n* = 122). Parents reported on school factors and HLD; pre-existing anxiety and co-occurring difficulties; anxiety, ADHD, and oppositional symptoms; demographics; and medications. Parents retrospectively reported more children often looked forward to school pre-pandemic, than during the pandemic. Anxiety symptoms, but not pre-existing anxiety disorder status, were associated with HLD after accounting for covariates. ADHD inattention symptoms were also associated with HLD. Results support recommendations to continue pre-pandemic supports to assist with ADHD symptoms during home learning, and strategies/supports for families are discussed.

**Supplementary Information:**

The online version contains supplementary material available at 10.1007/s10578-022-01338-3.

The COVID-19 pandemic has profoundly disrupted daily life for children and adolescents (henceforth ‘children’) and their families. Emerging literature suggests that clinical groups such as children with neurodevelopmental conditions, including Attention-Deficit/Hyperactivity Disorder (ADHD) and mental health challenges, may be more negatively impacted by the pandemic than peers without pre-existing conditions [1–3]. It is important to understand the experiences of specific clinical groups during the COVID-19 pandemic to enable healthcare, educational, and family services to identify and address critical needs, both during and beyond the pandemic [4].

School and learning present challenges for children with ADHD in the traditional school environment, relative to children without ADHD [5–7]. The core symptoms of ADHD include difficulty focusing, organisational difficulties, and learning task completion, which have all been shown to contribute to school-based difficulties [5, 8]. ADHD often co-occurs with other conditions such as anxiety, oppositional defiant disorder, Autism Spectrum Disorder (ASD), and learning, speech, and language disorders, which often exacerbate school-based difficulties [9–12].

During the pandemic, children in Australia, and in many countries around the world, were required by the government to swiftly transition to learning remotely from home (henceforth ‘home learning’) unless they had an approved exemption, such as a parent working in essential healthcare. While it is likely all children have faced challenges related to this rapid and unexpected shift [2], this transition may have been particularly challenging for children with ADHD, with the in-person learning difficulties experienced by this population likely to extend to home learning [13], given challenges related to their environment and supports, transitions, and regulation. Whilst a body of emerging research is exploring the mental health impacts of the COVID-19 pandemic in children with ADHD [14–17], less research has focused on home learning specifically.

The small body of existing research on home learning experiences in children with ADHD suggests that children with ADHD and their families have experienced greater home learning difficulties (HLD) compared to children without ADHD during the pandemic [2, 13]. One study of home learning experiences in adolescents with and without ADHD (*N* = 238; ages 15–17) indicated that almost a quarter of the sample were reported to have not engaged in any home learning during the survey period. Of the adolescents who had engaged in home learning, almost three-quarters spent an average of three or fewer total hours of schoolwork on school days; below the recommended quantity to learn new material and reinforce material learnt previously (3–4 h of learning activities for grades 7–12 students in United States guidance) [18], which may be especially important for students with learning challenges [13]. A study of children with neurodevelopmental disorders (*N* = 49; ages 6–17 years) and caregivers in rural United States showed that most were not receiving the recommended quantity of direct online instruction; and that the majority of support services (e.g., school counselling, speech therapy) had been discontinued during home learning [2].

HLD were also reported in a survey of parent perceptions of home learning experiences among three combined samples, comprised of children and adolescents with and without ADHD from the United States, and the Australian sample used in this study (*N* = 606; ages 6–17 years) [19]. Study results indicated that home learning challenges were largely consistent across the samples. Frequently reported difficulties included child-related (e.g., staying motivated and on-task), parent-related (e.g., balancing home learning with their work responsibilities) and teacher-related (e.g., poor instructional quality) challenges, highlighting multiple support needs relevant to this population during periods of home learning. To date, limited research has examined the predictors of HLD in children with ADHD.

Understanding factors predictive of HLD in children with ADHD may aid efforts to identify at-risk children requiring support both during- and post-pandemic, as well as potential protective factors. The aforementioned study of children with neurodevelopmental disorders and caregivers in rural United States (*N* = 49; ages 6–17 years) found that higher adaptive coping skills in the children were associated with greater total daily schoolwork completed, while parent employment was associated with more HLD [2]. In that sample, higher levels of emotional dysregulation in the children and parental mental health difficulties were asscociated with more HLD and less engagement. In another study, confidence levels of parents of children with ADHD in managing home learning was shown to be lower than that of parents of children without ADHD [13]. Inconsistent findings have been reported regarding the association between HLD and individualised education/school accommodation plans, designed to support interventions and accommodations for children with learning difficulties. Parents in one study of adolescents with both ADHD and a learning/support plan had more difficulties supporting home learning and home-school communication than parents of adolescents with ADHD without a plan [13]. By contrast, another study did not find an association between HLD and learning/support plan status [2].

Another factor that has been inconsistent in these studies is the association between HLD and socio-economic status. Family income was not associated with home learning experiences in the rural study of children with neurodevelopmental disorders [2]; whereas in another study of adolescents with and without ADHD [13], adolescents from low income families were more likely to report no online learning and low engagement in online class meetings, relative to adolescents from high income families. These results suggest socio-economic status may influence HLD, but this relationship requires further examination. Discontinued pre-existing supports during the pandemic may also make it difficult for children to benefit from strategies used previously to aid their learning and academic achievement, e.g., schoolwork extensions and modifications, tutoring, meals, and therapy [2].

Although yet to be examined, it is possible that co-occurring difficulties such as anxiety may also contribute to the HLD children with ADHD are experiencing during the pandemic. Previous research has shown that up to 50% of children with ADHD meet criteria for one or more anxiety disorders [9, 20, 21], the presence of which has been shown to exacerbate functional difficulties beyond those experienced by children with ADHD alone [21–24]. The presence of anxiety in addition to ADHD may increase distractibility and attentional difficulties [25, 26]. With the considerable routine changes and heightened uncertainty caused by COVID-19 [27], learning difficulties in children with ADHD may be exacerbated by anxiety symptoms, though this is yet to be examined. Understanding relationships between home learning experiences in children with ADHD and anxiety, alongside other clinical factors such as co-occurring ASD and symptoms of oppositionality, learning, language, speech disorders, as well as and other covariates such as SES, may assist in identifying individuals with ADHD who are most vulnerable to HLD and inform targeted supports for these children and families.

The purpose of the current study was to examine home learning experiences in children with ADHD during the COVID-19 pandemic, and the association between anxiety and HLD. Understanding learning arrangements and experiences for this population was anticipated to give background context to the examination of anxiety and HLD in children with ADHD. HLD was indicated by a combination of parent and child difficulties, as well as parent confidence in facilitating home learning. Specifically, in a sample of children with ADHD aged 5–17 years during the COVID-19 pandemic, this study aimed to:

1. Describe home learning experiences, including learning arrangements (e.g., school classes offered and attended online during survey period), quantity and timing of schoolwork and attention, and enthusiasm for school and home learning, before and during the pandemic.
2. Examine the association between anxiety (i.e., concurrent anxiety symptom severity, and pre-existing anxiety disorder status) and HLD. It was hypothesized that anxiety symptom severity, as well as pre-existing anxiety disorder status (present/not present), would be associated with HLD scores, before and after adjusting for age, gender, learning/speech/language disorders, ASD, medication use, oppositional and ADHD symptoms, and socio-economic status.

## Material and Methods

### Study Design

This study used baseline data from the ADHD COVID-19 Survey, a longitudinal study completed by parents of children aged 5–17 years with ADHD during the COVID-19 pandemic [17]. The first COVID-19 case was identified in Australia in January 2020 [28]. During the four-week period of data collection during May 2020, COVID-19 infection rates were lower in Australia than many other high-income countries. Government social distancing restrictions were in place, requiring children in most Australian states to learn from home unless they had an approved exemption (e.g., parent working in essential healthcare), although the precise duration of school closures varied throughout Australia [29, 30] (further contextual details contained in [17]).

### Participants

Two hundred and twenty-one families consented to participate in the study. Eligible parents were living in Australia, were at least 18 years old, and had a child aged 5–17 years at baseline who had been diagnosed with and/or treated for ADHD. For families with more than one child with ADHD, parents completed the survey about their eldest child within the age range. Participants were included in analyses if they provided data on all three measures of interest: home learning difficulties (HLD), anxiety symptoms and pre-existing anxiety disorder measures (*n* = 122), which meant that families who had not engaged in any home learning were not included. There were no systematic differences between those included and excluded from the sample on key characteristics (see Supplementary Table 1).

The average age of children in the included sample was 10.75 years (*SD* = 3.04) and 74.6% were male (see Table 1). Over 60% of children were reported to have a pre-existing anxiety disorder and almost 90% were taking medication, with 67.2% taking two or more medications. Almost three-quarters (72.0%) of children in the sample were reported to have one or more co-occurring condition, including ASD (18.2%), Oppositional Defiant Disorder (17.4%), and Conduct Disorder (0.8%), and more than one third (36.0%) had two or more co-occurring conditions. Almost all parent respondents were mothers (99.2%), and most had completed high school (90.2%).

**Table 1:** Sample characteristics

Child characteristics^a^	*n*	%
Age, *M* (*SD*), range	10.75 (3.04), 6–17	
Gender, *n* (%)		
Male	91	74.59
Female	31	25.41
ADHD inattention symptoms, *M* (*SD*)	18.57 (6.04)	
ADHD hyperactivity/impulsivity symptoms, *M* (*SD*)	14.10 (6.96)	
Oppositional symptoms, *M* (*SD*)	11.82 (6.54)	
Anxiety symptoms, *M* (*SD*)	9.93 (5.38)	
Pre-existing comorbidities, *n* (%)		
Anxiety	75	61.48
Autism spectrum disorder	22	18.18
Oppositional defiant disorder	21	17.36
Depression	13	10.74
Learning/Speech/Language disorder	12	9.84
Obsessive–Compulsive disorder	9	7.44
Tourette’s Syndrome/Tics	6	5.00
Conduct disorder	1	0.83
School grade, *n* (%)		
Preparatory/Foundation to Grade 6	77	63.11
Grade 7 to > Grade 12	45	36.89
Taking medication, *n* (%)	107	87.70
Stimulant/non-stimulant medication	100	81.97
Antidepressant medication	27	22.69

Parent characteristics^b^		
Age, *M* (*SD*), range	42.99 (5.40), 27–54	
Gender, *n* (%)		
Male	1	0.82
Female	121	99.18
Born in Australia, *n* (%)	95	81.20
Aboriginal or Torres Strait Islander, *n* (%)	2	1.64
High school completed, *n* (%)	110	90.16
Qualification completed, *n* (%)		
Trade certificate, diploma, or apprenticeship	37	30.33
University degree	78	63.94
Single parent, *n* (%)	26	21.31
Biological parent of child, *n* (%)	121	99.18
In paid employment prior to COVID-19 pandemic, *n* (%)	92	76.03
Casual	7	7.61
Part time	44	47.83
Full time	41	44.57
Any changes to work situation since pandemic, *n* (%)	45	48.91

Family characteristics^a^		
State of Australia, *n* (%)		
Victoria	41	33.61
New South Wales	38	31.15
Western Australia	19	15.57
Australian Capital Territory	18	14.75
South Australia	6	4.92
Queensland, Tasmania, Northern Territory	0	0.00
SEIFA Disadvantage score, *M* (*SD*), range	1038.94 (50.75), 879–1128	
Main language spoken at home is English, *n* (%)	119	97.54
Number of children living in home, *M* (*SD*), range	2.13 (0.81), 1–4	

*ADHD* Attention-Deficit/Hyperactivity Disorder

^a^*n* ranges 119–122

^b^*n* = 117–122

### Procedure

Ethical approval was obtained from Deakin University (HEAG-H 60_2020). This study was advertised by Australian ADHD organisations and support groups, who emailed the advertisement to families and posted it on their social media sites. Interested parents read the participant information and provided informed consent to participate in a longitudinal online survey. Data were collected and managed using the Research Electronic Data Capture (REDCap) software platform [31, 32].

### Measures

#### Sample Characteristics

Parents reported on child characteristics (i.e., age, gender, school grade and co-occurring conditions), and on their individual and family characteristics (i.e., age, gender, area of residence, country of birth, Aboriginal or Torres Strait Islander status, language spoken at home, relationship to the child, partner status, number of children at home ≥ 50% of the time, education and qualifications completed, pre-COVID-19 employment status and changes to work situation since COVID-19). Study-designed questions captured the child’s current medications, and were collapsed into an antidepressant medication (e.g., fluoxetine, sertraline, escitalopram; yes/no), and a stimulant and/or non-stimulant medication for ADHD (methylphenidate, lisdexamfetamine, dexamphetamine, guanfacine, atomoxetine; yes/no). The Socio-Economic Indexes for Areas (SEIFA) is presented for participants, which ranks areas of Australia according to the relative level of socio-economic disadvantage based on geographical area [33]. SEIFA scores are standardised (*M* = 1,000, *SD* = 100), where higher scores indicate relative socio-economic advantage.

#### Anxiety Disorders and Other Co-occurring conditions

Parents reported (yes/no) whether their child had been diagnosed with any of the following: anxiety disorders, depressive disorder, ASD, Oppositional Defiant Disorder, Conduct Disorder, Obsessive–Compulsive Disorder, learning/speech/language disorders, and Tourette’s Syndrome/Tics.

#### Anxiety Symptoms

Parents reported their child’s anxiety symptoms using the Brief Spence Children’s Anxiety Scale – Parent Report (SCAS-P-8) [34]. This 8-item measure (α = 0.87) assesses anxiety symptoms during the past two weeks, including generalised, social, and separation anxiety, as well as panic/agoraphobia, and is a brief version of the Spence Children’s Anxiety Scale [35]. The brief measure has been shown to have acceptable-to-good internal consistency, agreement among reporters, and good convergent and divergent validity with the full-length scale [34, 35]. Items (e.g., “Worries something bad will happen to him/her”) are rated on a four-point scale from “never” to “always”, with the total score reflecting the sum of the scores, ranging between 0–24, where higher scores indicate greater severity of anxiety symptoms.

#### ADHD and Oppositional Symptoms

The Swanson, Nolan and Pelham Rating Scale (SNAP-IV) [36] is a 26-item scale used to assess symptoms of inattention (ADHD-I; 9 items; α = 0.91), hyperactivity/impulsivity (ADHD-H; 9 items; α = 0.91) and oppositional behaviour (8 items; α = 0.93) in children aged 6–17 years. Items were rated on a 4-point scale to estimate the severity of symptoms during the previous two-week period from “not at all” to “very much” by parents (e.g., “Often has difficulty sustaining attention in tasks or play activities”). Items are summed for each subscale, with higher scores reflecting greater symptom severity [37]. An evaluation showed the SNAP-IV has good reliability (α = 0.94) and parent ratings were significantly higher for children who met ADHD criteria on a diagnostic ADHD measure than for those who did not [38].

#### School and HLD

Parents completed home learning questions if they reported that their child was school age, had stopped attending school in person or had participated in any online learning in the past fortnight. Questions were from the Home Adjustment to COVID-19 Scale (HACS) [39] (as used in [2, 13, 19]) and assessed home learning experiences of children with ADHD during the COVID-19 pandemic. Questions related to the child looking forward to school (pre-pandemic) and home learning during the pandemic, status of the child’s schooling (e.g., had they stopped attending in-person classes, were classes running online, were they engaging) and availability of classes, the amount of home learning children completed on school days, the best time of day the child attends to learning, and resources provided by schools. For example, “On average, how much school work (e.g., reading, project work, math) is your child doing at home on school days?”.

Difficulties experienced by children and their families in the two weeks prior to survey completion were also calculated using the HACS [39] (Total HLD score; α = 0.91; possible range 26–110; where higher scores indicated greater HLD). Three home learning subscales were summed, measuring: (1) difficulties experienced by the child (e.g., “focusing on their work”; 6 items on 5-point scale from “never” to “always”; α = 0.90, range 6–30); (2) level of parent confidence in helping their child (e.g., to “manage frustration”; 6 items on 5-point scale from “not at all confident” to “very confident”; α = 0.92, range 6–24); and, (3) level of parent difficulty (e.g., “understand your child’s learning assignments”; 11 items on 4-point scale from “not at all difficult” to “very difficult” items; α = 0.86, range 11–44), plus three single items that measured how enjoyable had it been for both: (1) the parent and (2) the child having schooling moved to home-based learning; as well as, (3) how challenging it had been for the child to have their schooling moved to home-based learning [39]. Subscale scores were calculated for participants who had responded to at least half of the subscale items (maximum missing data was a single item on any subscale) and missing data were replaced by an average of the existing items [as was done in 13]. Since higher parent confidence scores indicated greater confidence, scores were reversed to calculate total HLD scores [as was done in 13].

#### Statistical Analyses

Analyses were conducted using Stata 16. Descriptive statistics were computed to describe the characteristics of children, parents, and partners. T-tests and chi-square tests were used to examine differences between participants included and excluded from analysis. Home learning experiences were described (aim 1) including percentage of children who looked forward to going to school (pre-pandemic) and to home learning (during the pandemic), arrangements for classes and resources, quantity and time of schoolwork completed and time of day when child appears to best attend to schoolwork. Fisher’s exact test was used for between-groups analysis of reported hours of schoolwork completed for primary versus secondary students. Associations between anxiety symptom severity and HLD scores and pre-existing anxiety disorder status (i.e., present or not) with HLD scores (aim 2) were examined using unadjusted and adjusted linear regression analyses. Adjusted models accounted for ADHD inattention and hyperactivity/impulsivity symptoms, medication use, age, socio-economic index, oppositional symptoms, gender, ASD and learning/speech/language disorder status. Correlation coefficients between variables are also reported. Variance inflation factor and tolerance diagnostic statistics indicated no evidence of multicollinearity. A sensitivity analysis was also conducted, excluding HLD items overlapping with ADHD-I items (2 items from both the child difficulties and parent confidence HLD subscales, i.e., “focusing on their work”, “finishing their work”; 4 items total).

## Results

### Home Learning During the COVID-19 Pandemic

Parents reported retrospectively that on most days prior to the pandemic, 51.6% (*n* = 63) of children with ADHD looked forward to going to school, compared to parents reporting that 21.3% (*n* = 26) of children had been looking forward to home learning on most days during the pandemic. Furthermore, prior to the pandemic, 20.5% (*n* = 25) of children with ADHD rarely or never looked forward to going to school, compared to 50.8% (*n* = 62) who rarely or never looked forward to home learning during the pandemic.

Table 2 provides home learning background information for children with ADHD during the COVID-19 pandemic. Most had stopped attending school in-person (80.3%) and most reported that school classes were running online (88.3%). Parents reported that most children were engaging in at least some online learning (85.3%), though the average duration of online classes available per day was less than two hours (*M* = 1.65 h, *SD* = 1.87). Parents reported on the average hours of schoolwork their child was completing at home on school days, with a quarter (24.6%) completing zero or less than one hour, and half (50.8%) completing 2–4 h. The average hours of schoolwork completed at home on school days did not differ between primary and secondary students (*p* = .090). Most parents (77.5%) reported the time of day when their child attends best to home learning was the morning, and most reported their child completed most of their work at home in the mornings (61.3%). Most parents reported receiving home learning materials from their child’s school via online portals (83.6%).

**Table 2:** Summary of home learning characteristics for children with ADHD

Home learning characteristics	*n*	%
School classes during the prior two weeks		
Had stopped attending in-person classes at school^a^, *n* (%)	98	80.33
School classes had been running online^b^, *n* (%)	83	88.30
Engaging in any online learning^a^, *n* (%)	104	85.25
Hours of online classes available per day^c^, *M* (*SD*), range	1.65 (1.87), 0–7	
Average hours of schoolwork completed at home on school days^a^, *n* (%)		
0	8	6.56
< 1	22	18.03
2	18	14.75
3	21	17.21
4	23	18.85
5	12	9.84
6	14	11.48
> 6 h	4	3.28
Time of day child attends to home learning best^a^, *n* (%)		
Morning	93	77.50
Middle of the day	19	15.83
Afternoon	7	5.83
Evening	1	0.83
Time of day child completes most of their work at home^a^, *n* (%)		
Morning	73	61.34
Middle of the day	30	25.21
Afternoon	9	7.56
Evening	7	5.88
Format of distance learning materials provided by school^a^, *n* (%)		
Hard copy (i.e. mailed, sent home)	28	22.95
Online via virtual school portal	102	83.61
Online using existing platforms (i.e. YouTube)	26	21.31
Email	46	37.70
No learning materials provided	0	0.00
Other	8	6.56

^a^*n* ranges 119–122

^b^ *n* = 94

^c^*n* = 82

### Anxiety Symptoms and Home Learning Difficulties (HLD) Scores

Details of HLD measure scores for this sample (*M, SD*) are provided in Supplementary Table 2 and correlations between variables of interest can be found in Supplementary Table 3.

There was a significant positive association between anxiety symptoms and HLD scores in unadjusted and adjusted models,^1^ with anxiety symptom severity accounting for 4.3% of the unadjusted variation in HLD scores. The adjusted model explained 47.7% of variance in HLD scores, with ADHD-I symptoms the only other variable uniquely associated with anxiety symptoms in the adjusted model; see Table 3.

**Table 3:** Hierarchical regression examining association between anxiety symptoms and other predictors of HLD scores in children with ADHD

Predictors	*b* [*95% CI*]	*β*	*SE*	*t*	*p*	*R*^*2*^	*Adj. R*^*2*^
Model 1						0.04	0.04
Anxiety symptoms	0.55 [0.08, 1.01]	0.21	0.23	2.33	.022		
Model 2					< .001	0.48	0.43
Anxiety symptoms	0.41 [0.02, 0.81]	0.16	0.20	2.06	.042		
Gender	1.68 [− 3.04, 6.39]	0.05	2.38	0.71	.482		
Child age	− 0.51 [− 1.27, 0.25]	0.02	0.38	− 1.33	.185		
Learning/speech/language disorder	0.79 [− 6.15, 7.73]	− 0.11	3.50	0.23	.822		
Medication use	1.28 [− 4.48, 7.05]	0.04	2.91	0.44	.659		
SEIFA	− 0.03 [− 0.08, 0.01]	− 0.13	0.02	− 1.67	.097		
Oppositional symptoms	0.00 [− 0.39, 0.40]	0.00	0.20	0.03	.980		
ADHD-I symptoms	1.72 [1.27, 2.16]	0.74	0.22	7.64	< .001		
ADHD-H symptoms	− 0.33 [− 0.79, 0.13]	− 0.16	0.23	− 1.42	.158		
ASD diagnosis	1.64 [− 3.96, 7.24]	0.04	2.82	0.58	.563		

*n* = 119 in adjusted model

*ADHD-I* attention-deficit/hyperactivity disorder inattention symptoms, *ADHD-H* attention-deficit/hyperactivity disorder hyperactive/impulsive symptoms, *ASD* Autism Spectrum Disorder, *HLD* home learning difficulties, *SEIFA* Socio-Economic Indexes for Areas

We also examined whether results were consistent when using subscales of HLD (i.e., child difficulties, parent difficulties and parent confidence subscales) as outcome variables (see Supplementary Tables 4–6). A similar pattern of results was revealed. Anxiety symptoms were uniquely positively associated with the parent difficulties subscale, but not the child difficulties or parent confidence subscales, in adjusted analyses. ADHD-I symptom severity was the only other variable uniquely associated with the HLD subscales in all three adjusted models.

Pre-existing anxiety disorder status was not associated with HLD in unadjusted or adjusted models; see Table 4. The adjusted model explained 45.99% of the variance in HLD scores, with the only significant association being with ADHD-I symptoms.

**Table 4:** Hierarchical regression examining the association between pre-existing anxiety disorder status and other predictors of HLD scores in children with ADHD

Predictors	*b [95% CI]*	*β*	*SE*	*t*	*p*	*R*^*2*^	*Adj. R*^*2*^
Model 1						0.01	0.01
Anxiety disorder status	3.36 [− 1.82, 8.55]	0.12	2.62	1.28	.202		
Model 2					< .001	0.46	0.41
Anxiety disorder status	1.85 [− 2.68, 6.38]	0.06	2.29	0.81	.421		
Gender	1.08 [− 3.67, 5.83]	0.03	2.40	0.45	.652		
Child age	− 0.43 [− 1.20, 0.34]	− 0.09	0.39	− 1.11	.270		
Learning/speech/language disorder	0.02 [− 7.01, 7.06]	0.00	3.55	0.01	.995		
Medication use	0.10 [− 6.10, 6.29]	0.00	3.13	0.03	.976		
SEIFA	− 0.03 [− 0.07. 0.01]	− 0.11	0.02	− 1.44	.154		
Oppositional symptoms	0.09 [− 0.29, 0.48]	0.04	0.20	0.48	.635		
ADHD-I symptoms	1.70 [1.25, 2.15]	0.73	0.23	7.44	< .001		
ADHD-H symptoms	− 0.29 [− 0.75, 0.18]	− 0.14	0.23	− 1.22	.225		
ASD diagnosis	2.28 [− 3.39, 7.96]	0.06	2.86	0.80	.427		

*n* = 119 in adjusted model

*ADHD-I* attention-deficit/hyperactivity disorder inattention symptoms, *ADHD-H* attention-deficit/hyperactivity disorder hyperactive/impulsive symptoms, *ASD* Autism Spectrum Disorder, *HLD* home learning difficulties, *SEIFA* Socio-Economic Indexes for Areas

## Discussion

This study described the home learning experiences of a sample of Australian children with ADHD, and associations between anxiety and HLD during COVID-19-related restrictions. Parents retrospectively reported that, pre-COVID-19, more children looked forward to school compared to during the pandemic. Parents also perceived children to be less engaged in schoolwork during COVID-19 home learning relative to engagement at school pre-pandemic, which may suggest that children learning from home are spending less time engaging with both new content and reinforcing old content; however, there does not appear to be a set Australian standard for the quantity of learning activities on school days. Results of the current study are consistent with a prior study that reported children in their sample received less than two hours of direct instruction from teachers during home learning, with most children completing an average of only 0–3 h of schoolwork on school days [13]. Longitudinal studies are needed to evaluate whether reduced direct teacher instruction, time engaged in schoolwork, and/or the duration of home learning, have an impact on later learning and academic achievement among children with ADHD. It is likely that there have been a range of durations in home learning, which may impact the difficulties experienced.

In the current sample, there was a unique association between anxiety symptom severity, but not pre-existing anxiety disorder status, and HLD. Anxiety symptom severity was positively associated with HLD, before and after adjusting for covariates. However, there was no association between anxiety disorder status and HLD in unadjusted or adjusted analyses. Symptoms of oppositionality or other co-occurring domains were not associated with HLD. Individuals with mental health difficulties such as anxiety have been reported to be more impacted by COVID-19-related difficulties than peers without mental health difficulties [1, 40]. The results of the current study suggest that anxiety symptom severity may be one important indicator of HLD and has the potential to exacerbate school and learning difficulties in children with ADHD. It is therefore important that anxiety symptoms are identified and addressed as early as possible in children with ADHD to minimise ongoing symptoms and impacts on home learning. Parents may support children by acknowledging the changes they have experienced, providing contained opportunities to discuss and validate their child’s concerns, maintaining healthy family routines, managing their own concerns and modelling helpful coping, as well as involving clinicians and additional supports as required to reduce the impact of symptoms [41–43]. Due to the measures employed, it was not possible in this study to examine the role of specific types of anxiety in predicting HLD, relative to anxiety overall, however this could be explored in future research. For example, it is plausible that children with social or separation anxiety may have experienced fewer anxiety-provoking in-person social encounters and less separation during periods of home learning, relative to in-person learning. This may potentially offer insights into associations between various presentations of anxiety and HLD and more specific risk factors.

The results of this study also point to the importance of continued supports to help with ADHD symptoms during home learning to optimise educational outcomes. Results suggest that ADHD-I symptoms were more strongly linked to HLD than anxiety, ADHD-H symptoms or other co-occurring factors examined in this study. This is consistent with previous research that found ADHD-I symptoms to be more strongly associated with academic difficulties compared to ADHD-H symptoms [44]. Although statistical testing indicated multicollinearity was not problematic, it is possible that the measurement of HLD, particularly in the child difficulties domain, captured symptoms of inattention (e.g., maintaining focus and interest) to a greater extent than symptoms of hyperactivity/impulsivity, which may contribute to explaining why ADHD-I, but not ADHD-H symptoms were associated with HLD items. A sensitivity analysis that excluded HLD items that overlapped with ADHD-I items produced consistent results. It is also plausible that ADHD-H symptoms may be less problematic in the context of home learning; for example, if children have greater opportunities for schedule flexibility or movement breaks, without disrupting others as they may within an in-person classroom setting. Regardless, these findings reinforce the importance of the ADHD management guidelines during the COVID-19 pandemic, which recommended that where possible, children with ADHD continue their usual treatments to help with ADHD symptoms during the pandemic [45]. ADHD-I symptoms were also uniquely associated with the HLD subscales measuring parent difficulties and parent confidence with home learning.

Despite almost 88% of children in this sample taking one or more medications during the examined period, children with ADHD were still experiencing substantial HLD. This suggests that in addition to pharmacological interventions, children with ADHD and their families are likely to benefit from other strategies to assist with home learning. Established strategies routinely recommended for children with ADHD at school (e.g., a quiet, uncluttered work area free from distractions, regular rest breaks, repeating instructions to ensure comprehension, visual checklists/schedules, task breakdown) [46–48], applied to the home setting, could be helpful. Additionally, use of accommodations that specifically target organisational skills in an online learning context may also be beneficial.

Strategies parents have reported as effective in supporting home learning during COVID-19 include limiting environmental distractions, supporting their child with understanding and planning their work, assistance with time management, exercise, and regular breaks from learning [19]. However, it is also important for educators and clinicians to keep in mind that some strategies (e.g., quiet work area) may be difficult or impossible to implement for some families, depending on their housing, family composition and their work-related demands. It is important when working with families before and during home learning contexts for clinicians and educators to assess which strategies are most suited to the family context and whether modification of some approaches is required to facilitate improved home learning outcomes. Given the heterogeneity of ADHD symptom profiles and that this study found symptoms of inattention, but not hyperactivity/impulsivity, to be associated with HLD, the specific challenges an individual child experiences, as well as feasibility, should be considered when implementing support strategies and tailored to their needs.

Socio-economic status was not uniquely associated with HLD in the current study. This finding is consistent with an earlier study of children and adolescents [2], but contrasts with another study in which adolescents from lower income families were more likely than those from higher income families to receive no online learning and less likely to engage in online class meetings [13]. It is possible that the neighbourhood-level socio-economic indicator used in the current study was not as sensitive as a family-based indicator of socio-economic status may have been.

Results of the current study support the idea that in addition to supporting children with ADHD, it is important that parents are assisted with home learning supports and strategies in adapting to the challenge of facilitating and supporting learning from home [13]. Poor child engagement and enthusiasm in home learning may compound pressure on parents, who are often expected to facilitate completion of schoolwork, despite reductions in their usual supports (e.g. social and family support, childcare). In this sample, over half of the parents reported their child rarely or never looked forward to home learning during the pandemic, compared to one in five who rarely or never looked forward to going to school pre-COVID-19. Additionally, the percentage of children looking forward to home learning on most days (one in five) was lower than those who looked forward to going to school on most days pre-pandemic (over half). In this study, more than half of the children completed less than three hours of schoolwork per day, and the whole sample reported receiving an average of less than two hours of direct instruction per school day. These results are suggestive of poor engagement in home schooling and speak to the need for increased parental and/or teaching support during home learning. It would be useful to explore whether parents were attempting to encourage children to complete more than the reported hours completed, whether children were able to complete their allocated schoolwork within this time, as well as considering impacts of parent mental health, which predicted less engagement and greater HLD in a US sample [2].

Supplementary home learning supports for parents of children with ADHD may include more regular direct contact with teachers and school support staff, with the aim of promoting child engagement. Moreover, strategies previously deemed effective by classroom teachers could be discussed with parents, with many children with ADHD having lost access to the additional learning supports they previously received [2, 13]. Children with ADHD may experience ongoing challenges during and beyond the pandemic and will require supports to minimise the academic and clinical impacts of the pandemic. Re-opening and keeping schools and early childhood education and care facilities open during and beyond the current pandemic has been identified as a priority to minimise the disruptions that disproportionately affect students with pre-existing difficulties [49, 50]. Preventing and/or promoting smooth transitions to future periods of home learning in the context of evolving COVID-19 variants and potential future pandemics and associated school closures is important to minimise negative consequences to children [50, 51]. It would be useful to continue to examine outcomes for children with ADHD during the pandemic to facilitate early identification of difficulties and enable a prompt response. These may include, for example, additional academic support such as more regular online contact with teachers, and referral to clinical services to review support plans. In supporting students’ home learning, parents are also likely to benefit from employers and policies supporting family-friendly, flexible workplace arrangements.

While there are data available detailing strategies parents have found helpful during home learning and recommendations for parent-teacher collaboration [e.g. 19, 52, 53], assumptions cannot be made about the support needs of parents and teachers during home learning and return to in-person learning. Although the current study examined HLD in children with ADHD, previous research reported that parental experiences of home learning did not significantly differ by ADHD diagnosis (present/not present by parent report), country (United States compared to Australia), or child age [19]. This may suggest parental experiences of home learning may be universal, with research highlighting the indication for supports. Transitioning back to school may be particularly challenging for children who have experienced disruptions to learning, and those who experience learning challenges. Although suggestions for parents about how to promote a successful return to school are emerging [e.g., 50, 54, 55], this needs to be empirically evaluated in the context of COVID-19, including asking parents, teachers, and children directly about their concerns and needs [56].

Findings of the current study also support the notion that enhanced home learning models are needed, particularly for students with differing learning needs, including children with ADHD. With online learning environments forecast to have an increasing place in our educational systems into the future [57, 58], opportunities exist for future research to explore implementation and evaluation of strategies to support children in online learning contexts. Home learning systems differ across schools and contexts; efforts aimed at optimising the quality of education provided in this format, such as consideration of systems that scaffold time management and most importantly maintain student engagement, are likely to be useful beyond the current pandemic context, particularly for students with ADHD and other conditions, which may impact on learning [50, 51].

Findings from the current study should be interpreted in the context of several limitations. The availability of parent-reported data only may be a limitation, given possible reporting discrepancies with children’s perceptions [59]. Future research should endeavour to examine child perceptions in future investigations of the relationships explored in this study, particularly for internalizing domains such as anxiety, which was not feasible within the current study. Further, the special education status and nature of additional supports for this sample before and during home learning were not known but may also impact the degree of HLD and could be considered with future research. Our self-selected sample of parents may not be representative of all children and adolescents with ADHD due to selection criteria, however, a comparison between our sample characteristics and a large clinical audit of Australian children (*N* = 1528; aged 3–19 years) with ADHD attending paediatric services revealed similar characteristics, with the exception of higher levels of comorbid depression and anxiety in the current sample [60]. Therefore, further research in this population is warranted to replicate the study results and enhance generalizability. Inclusion criteria relied on parent report; researchers were unable to independently confirm ADHD diagnostic status. Retrospective recall of some parent-reported data (e.g., how often their child appeared to look forward to school pre-pandemic) may be prone to reporting biases. Cross-sectional analyses limit the extent to which prediction of HLD can be made, with longitudinal follow-up warranted. The lack of a comparison group of children without ADHD limits the extent to which we can attribute findings specifically to ADHD. It is possible that anxiety and ADHD-I symptoms may also be associated with HLD in the general population or in children with other mental health conditions. Future research, including broader samples of children, could address this question.

To our knowledge, this represents one of the first Australian studies of home learning experiences among children with ADHD and their parents during the COVID-19 pandemic with a focus on anxiety. It documents the numerous challenges faced by children with ADHD during a time of rapid change and provides preliminary evidence of the need for adequate and timely supports from schools, policymakers, and healthcare services as impacts of the pandemic continue to evolve and longer-term effects become more evident. This is underscored by the fact that many children with ADHD already experienced challenges at school prior to the COVID-19 pandemic. Supports need to be multi-faceted and tailored to the specific needs of children with ADHD, their parents, and their teaching communities.

## Summary

The COVID-19 pandemic has impacted children and adolescents around the world, particularly those with pre-existing conditions, including attention-deficit/hyperactivity disorder (ADHD) and anxiety. This is one of the first studies to examine home learning experiences in children and adolescents (age 5–17 years; *n* = 122) with ADHD in Australia during COVID-19 social restrictions, and the role of anxiety in understanding HLD. We found that anxiety symptom severity, but not pre-existing anxiety disorder status, was robustly associated with HLD, before and after adjusting for a range of other clinical factors. ADHD-I symptom severity was the only other variable uniquely associated with HLD. This suggests that strategies to support anxiety and ADHD-I symptoms may be helpful for children with ADHD during home learning. The results reinforce the recommendation that pre-pandemic ADHD symptom supports are continued during the pandemic, and that anxiety symptoms are identified and supported. Children and adolescents with ADHD, and their families, may benefit from additional strategies and supports during and beyond periods of home learning.

## Supplementary Information

Below is the link to the electronic supplementary material.

Electronic supplementary material 1 (DOCX 41 kb)

## Footnotes

1 A sensitivity analysis was also conducted, excluding HLD items overlapping with ADHD-I items. Results were consistent (see Supplementary Tables 7–10).
