# Supplementary material for: Associations Between Anxiety and Home Learning Difficulties in Children and Adolescents with ADHD During the COVID-19 Pandemic
Source: Child Psychiatry Hum Dev. 2022 Mar 15;54(5):1347–59. doi: 10.1007/s10578-022-01338-3 (PMC8922068; doi:10.1007/s10578-022-01338-3)
Supplement: Supplementary file 1 — Electronic supplementary material 1 (DOCX 41 kb) [file 10578_2022_1338_MOESM1_ESM.docx]

**Supplementary Tables**

**Supplementary Table 1** Sample characteristics for participants included and excluded in analyses.

|  | Included^a^ | Excluded^b^ | *p* |
| --- | --- | --- | --- |
| *Child characteristics* |  |  |  |
| Age *M (SD), range* | 10.75 (3.04), 6-17 | 10.30 (3.17), 5-17 | .289 |
| Gender *n (%)*  Male  Female | 91 (74.59)  31 (25.41) | 73 (79.35)  19 (20.65) | .415 |
| *Parent and family characteristics* |  |  |  |
| Age *M (SD), range* | 42.99 (5.40), 27-54 | 41.87 (6.11), 28-56 | .149 |
| Gender *n (%)*  Male  Female  SEIFA Disadvantage score *M (SD), range* | 1 (0.82)  121 (99.18)  1038.94 (50.75), 879-1128 | 4 (4.04)  95 (95.96)  1038.41 (49.87), 922-1127 | .109  .938 |

*Note.* ^a^ *n*=119-122; ^b^ *n*=93-99. *p* values represent t-tests for continuous variables and chi-square tests for categorical variables.

**Supplementary Table 2** Summary of home learning difficulty (HLD) scores and subdomains in children with ADHD.

| Variable | *M* | *SD* | *Range* | α |
| --- | --- | --- | --- | --- |
| Child difficulties | 22.39 | 5.43 | 9-30 | 0.90 |
| Parent confidence | 13.72 | 4.72 | 6-24 | 0.92 |
| Parent difficulties | 25.41 | 6.97 | 11-44 | 0.86 |
| Total HLD | 72.82 | 14.12 | 34-102 | 0.91 |

Note. *n* = 122.

**Supplementary Table 3** Correlation coefficients between study variables

|  | Child age | SEIFA | ADHD-I | ADHD-H | Anxiety symptoms | Oppositional symptoms | HLD scores |
| --- | --- | --- | --- | --- | --- | --- | --- |
| Child age | - | - | - | - | - | - | - |
| SEIFA | 0.09 | - | - | - | - | - | - |
| ADHD-I | -0.06 | 0.07 | - | - | - | - | - |
| ADHD-H | -0.39* | -0.14 | 0.60* | - | - | - | - |
| Anxiety symptoms | 0.02 | 0.06 | 0.15 | 0.19* | - | - | - |
| Oppositional symptoms | -0.11 | -0.09 | 0.42* | 0.54* | 0.28* | - | - |
| HLD scores | -0.08 | -0.04 | 0.65* | 0.36* | 0.21* | 0.30* | - |

*Note.* * significant at *p* <.05. ADHD-I = attention-deficit/hyperactivity disorder inattention symptoms. ADHD-H = attention-deficit/hyperactivity disorder hyperactive/impulsive symptoms. HLD = home learning difficulties. SEIFA = Socio-Economic Indexes for Areas.

**Supplementary Table 4** Hierarchical regression examining association between anxiety symptoms and other predictors of child difficulties with home learning in children with ADHD

| Predictors | *b* [*95% CI*] | *β* | *SE* | *t* | *p* | *R^2^* | *Adj. R^2^* |
| --- | --- | --- | --- | --- | --- | --- | --- |
| Model 1  Anxiety symptoms | 0.17 [-0.01, 0.35] | 0.17 | 0.09 | 1.92 | .057 | 0.03 | 0.02 |
| Model 2  Anxiety symptoms  Gender  Age  Learning/speech/language disorder  Medication use  SEIFA  Oppositional symptoms  ADHD-I symptoms  ADHD-H symptoms  ASD diagnosis | 0.07 [-0.08, 0.22]  0.62 [-1.18, 2.43]  -0.20 [-0.49, 0.09]  0.69 [-1.97, 3.35]  0.13 [-2.08, 2.33]  -0.01 [-0.02, 0.01]  0.04 [-0.11, 0.19]  0.67 [0.50, 0.84]  -0.13 [-0.31, 0.04]  1.93 [-0.22, 4.07] | 0.07  0.05  -0.11  0.04  0.01  -0.07  0.05  0.75  -1.17  0.14 | 0.08  0.91  0.15  1.34  1.11  0.01  0.08  0.09  0.09  1.08 | 0.93  0.69  -1.37  0.52  0.11  -0.93  0.50  7.83  -1.48  1.78 | <.001  .354  .494  .174  .608  .910  .355  .617  <.001  .142  .077 | 0.48 | 0.44 |

Note. *n* = 119 in adjusted model. ADHD-I = attention-deficit/hyperactivity disorder inattention symptoms. ADHD-H = attention-deficit/hyperactivity disorder hyperactive/impulsive symptoms. ASD = Autism Spectrum Disorder. SEIFA = Socio-Economic Indexes for Areas.

**Supplementary Table 5** Hierarchical regression examining association between anxiety symptoms and other predictors of parent difficulties with home learning in children with ADHD

| Predictors | *b* [*95% CI*] | *β* | *SE* | *t* | *p* | *R^2^* | *Adj. R^2^* |
| --- | --- | --- | --- | --- | --- | --- | --- |
| Model 1  Anxiety symptoms | 0.23 [-0.00, 0.46] | 0.18 | 0.12 | 1.95 | .054 | 0.03 | 0.02 |
| Model 2  Anxiety symptoms  Gender  Age  Learning/speech/language disorder  Medication use  SEIFA  Oppositional symptoms  ADHD-I symptoms  ADHD-H symptoms  ASD diagnosis | 0.29 [0.04, 0.54]  1.24 [-1.70, 4.18]  -0.42 [-0.90, 0.05]  1.15 [-3.18, 5.47]  0.90 [-2.69, 4.50]  -0.01 [-0.04, 0.01]  -0.13 [-0.38, 0.11]  0.47 [0.19, 0.75]  -0.13 [-0.41, 0.16]  -1.00 [-4.50, 2.49] | 0.22  0.08  -0.18  0.05  0.05  -0.10  -0.12  0.41  -0.12  -0.05 | 0.13  1.48  0.24  2.18  1.81  0.01  0.12  0.14  0.14  1.76 | 2.31  0.84  -1.76  0.52  0.50  -1.10  -1.09  3.37  -0.88  -0.57 | .019  .023  .404  .081  .601  .620  .272  .279  .001  .382  .570 | 0.17 | 0.10 |

Note. *n* = 119 in adjusted model. ADHD-I = attention-deficit/hyperactivity disorder inattention symptoms. ADHD-H = attention-deficit/hyperactivity disorder hyperactive/impulsive symptoms. ASD = Autism Spectrum Disorder. SEIFA = Socio-Economic Indexes for Areas.

**Supplementary Table 6** Hierarchical regression examining association between anxiety symptoms and other predictors of parent confidence with home learning in children with ADHD

| Predictors | *b* [*95% CI*] | *β* | *SE* | *t* | *p* | *R^2^* | *Adj. R^2^* |
| --- | --- | --- | --- | --- | --- | --- | --- |
| Model 1  Anxiety symptoms | -0.17 [-0.32, -0.01] | -0.19 | 0.08 | -2.10 | .038 | 0.04 | 0.03 |
| Model 2  Anxiety symptoms  Gender  Age  Learning/speech/language disorder  Medication use  SEIFA  Oppositional symptoms  ADHD-I symptoms  ADHD-H symptoms  ASD diagnosis | -0.07 [-0.23, 0.08]  -0.00 [-1.80, 1.79]  -0.20 [-0.49, 0.09]  0.94 [-1.70, 3.59]  -0.03 [-2.23, 2.16]  0.01 [-0.00, 0.03]  -0.11 [-0.26, 0.04]  -0.33 [-0.50, -0.16]  -0.01 [-0.19, 0.16]  -0.79 [-2.92, 1.35] | -0.08  -0.00  -0.13  0.06  -0.00  0.13  -0.14  -0.43  -0.02  -0.06 | 0.08  0.91  0.15  1.33  1.11  0.01  0.08  0.09  0.09  1.08 | -0.97  -0.00  -1.35  0.71  -0.03  1.49  -1.40  -3.87  -0.15  -0.73 | <.001  .332  .996  .179  .482  .976  .139  .165  <.001  .882  .466 | 0.32 | 0.26 |

Note. *n* = 119 in adjusted model. ADHD-I = attention-deficit/hyperactivity disorder inattention symptoms. ADHD-H = attention-deficit/hyperactivity disorder hyperactive/impulsive symptoms. ASD = Autism Spectrum Disorder. SEIFA = Socio-Economic Indexes for Areas.

**Supplementary Table 7** Sensitivity analysis^a^ examining associations between anxiety symptoms and other predictors of HLD in children with ADHD

|  | *b* [*95% CI*] | *β* | *SE* | *t* | *p* | *R^2^* | *Adj. R^2^* |
| --- | --- | --- | --- | --- | --- | --- | --- |
| Overall Model Fit  Predictors  Anxiety symptoms  Gender  Age  Learning/speech/language disorder  Medication use  SEIFA  Oppositional symptoms  ADHD-I symptoms  ADHD-H symptoms  ASD diagnosis | 0.41 [0.07, 0.76]  2.00 [-2.07, 6.07]  -0.52 [-1.18, 0.14]  1.27 [-4.99, 7.52]  0.85 [-4.20, 5.89]  -0.03 [-0.07, 0.01]  -0.06 [-0.40, 0.28]  1.26 [0.88, 1.65]  -0.19 [-0.58, 0.21]  0.50 [-4.43, 5.42] | 0.19  0.08  -0.13  0.03  0.03  -0.13  -0.03  0.67  -0.00  0.02 | 0.17  2.05  0.33  3.15  2.54  0.02  0.17  0.19  0.20  2.48 | 2.38  0.97  -1.57  0.40  0.33  -1.67  -0.35  6.50  -0.94  0.20 | <.001  .019  .333  .120  .689  .740  .097  .724  <.001  .351  .842 | 0.43 | 0.38 |

Note. *n* = 119 in adjusted model. ADHD-I = attention-deficit/hyperactivity disorder inattention symptoms. ADHD-H = attention-deficit/hyperactivity disorder hyperactive/impulsive symptoms. ASD = Autism Spectrum Disorder. SEIFA = Socio-Economic Indexes for Areas. HLD = home learning difficulties. ^a^ Sensitivity analysis excluded 4 items from the HLD scale overlapping with inattention.

**Supplementary Table 8** Sensitivity analysis^a^ examining associations between pre-existing anxiety disorder and other predictors of HLD in children with ADHD

|  | *b* [*95% CI*] | *β* | *SE* | *t* | *p* | *R^2^* | *Adj. R^2^* |
| --- | --- | --- | --- | --- | --- | --- | --- |
| Overall Model Fit  Predictors  Anxiety disorder status  Gender  Age  Learning/speech/language disorder  Medication use  SEIFA  Oppositional symptoms  ADHD-I symptoms  ADHD-H symptoms  ASD diagnosis | 1.97 [-1.95, 5.89]  1.36 [-2.76, 5.48]  -0.45 [-1.12, 0.22]  0.44 [-5.94, 6.82]  -0.40 [-5.87, 5.07]  -0.03 [-0.06, 0.01]  0.03 [-0.30, 0.37]  1.25 [0.86, 1.65]  -0.15 [-0.56, 0.25]  1.19 [-3.81, 6.19] | 0.08  0.05  -0.12  0.01  -0.01  -0.11  0.02  0.66  -0.09  0.04 | 1.98  2.08  0.34  3.22  2.76  0.02  0.17  0.20  0.20  2.52 | 0.99  0.65  -1.34  0.14  -0.15  -1.39  0.19  6.31  -0.75  0.47 | <.001  .322  .514  .183  .891  .885  .169  .852  <.001  .453  .637 | 0.41 | 0.35 |

Note. *n* = 119 in adjusted model. ADHD-I = attention-deficit/hyperactivity disorder inattention symptoms. ADHD-H = attention-deficit/hyperactivity disorder hyperactive/impulsive symptoms. ASD = Autism Spectrum Disorder. SEIFA = Socio-Economic Indexes for Areas. HLD = home learning difficulties. ^a^ Sensitivity analysis excluded 4 items from the HLD scale overlapping with inattention.

**Supplementary Table 9** Sensitivity analysis^a^ examining associations between anxiety symptoms and other predictors of child difficulties with home learning in children with ADHD

|  | *b* [*95% CI*] | *β* | *SE* | *t* | *p* | *R^2^* | *Adj. R^2^* |
| --- | --- | --- | --- | --- | --- | --- | --- |
| Overall Model Fit  Predictors  Anxiety symptoms  Gender  Age  Learning/speech/language disorder  Medication use  SEIFA  Oppositional symptoms  ADHD-I symptoms  ADHD-H symptoms  ASD diagnosis | 1.97 [-1.95, 5.89]  1.36 [-2.76, 5.48]  -0.45 [-1.12, 0.22]  0.44 [-5.94, 6.82]  -0.4 [-5.87, 5.07]  -0.03 [-0.06, 0.01]  0.03 [-0.3, 0.37]  1.25 [0.86, 1.65]  -0.15 [-0.56, 0.25]  1.19 [-3.81, 6.19] | 0.07  0.07  -0.12  0.02  -0.04  -0.06  0.06  0.62  -0.06  0.13 | 0.05  0.66  0.11  1.01  0.82  0.01  0.05  0.06  0.06  0.80 | 0.88  0.83  -1.4  0.29  -0.4  -0.73  0.63  6.04  -0.48  1.63 | <.001  .379  .407  .165  .773  .690  .466  .533  <.001  .633  .106 | 0.41 | 0.36 |

Note. *n* = 119 in adjusted model. ADHD-I = attention-deficit/hyperactivity disorder inattention symptoms. ADHD-H = attention-deficit/hyperactivity disorder hyperactive/impulsive symptoms. ASD = Autism Spectrum Disorder. SEIFA = Socio-Economic Indexes for Areas. ^a^ Sensitivity analysis excluded 4 items from the HLD scale overlapping with inattention.

**Supplementary Table 10** Sensitivity analysis^a^ examining associations between anxiety symptoms and other predictors of parent confidence with home learning in children with ADHD

|  | *b* [*95% CI*] | *β* | *SE* | *t* | *p* | *R^2^* | *Adj. R^2^* |
| --- | --- | --- | --- | --- | --- | --- | --- |
| Overall Model Fit  Predictors  Anxiety symptoms  Gender  Age  Learning/speech/language disorder  Medication use  SEIFA  Oppositional symptoms  ADHD-I symptoms  ADHD-H symptoms  ASD diagnosis | 0.07 [-0.03, 0.17]  0.32 [-0.83, 1.46]  0.10 [-0.09, 0.29]  -0.40 [-2.09, 1.28]  0.00 [-1.40, 1.40]  -0.01 [-0.02, 0.00]  0.06 [-0.04, 0.16]  0.21 [0.10, 0.32]  0.02 [-0.09, 0.14]  0.46 [-0.91, 1.82] | 0.12  0.05  0.10  -0.04  0.00  -0.15  0.13  0.41  0.05  0.06 | 0.05  0.58  0.09  0.85  0.71  0.01  0.05  0.05  0.06  0.69 | 1.38  0.55  1.07  -0.47  0.00  -1.76  1.22  3.76  0.41  0.66 | <.001  .171  .586  .289  .637  .997  .081  .225  <.001  .682  .509 | 0.34 | 0.27 |

Note. *n* = 119 in adjusted model. ADHD-I = attention-deficit/hyperactivity disorder inattention symptoms. ADHD-H = attention-deficit/hyperactivity disorder hyperactive/impulsive symptoms. ASD = Autism Spectrum Disorder. SEIFA = Socio-Economic Indexes for Areas. ^a^ Sensitivity analysis excluded 4 items from the HLD scale overlapping with inattention.
